# Pediatric CNS relapse of neuroblastoma treated with upright proton craniospinal irradiation

**DOI:** 10.1016/j.ctro.2025.101080

**Published:** 2025-11-19

**Authors:** Philip Blumenfeld, Alexander Pryanichnikov, Zelig Tochner, Aaron M Allen, Iris Fried, David Gozal, Sean Marzeeq, Stéphane Ledot, Shimshon Winograd, Ayman Salhab, Marc Wygoda, Yair Hillman, Jon Feldman, Aron Popovtzer

**Affiliations:** a Sharett Institute of Oncology, Hadassah Medical Center, Hebrew University of Jerusalem, Jerusalem, Israel; b Institute of Biomedical Engineering, Karlsruhe Institute of Technology (KIT), Karlsruhe, Germany; c Division of Biomedical Physics in Radiation Oncology, German Cancer Research Center (DKFZ), Heidelberg, Germany; d Department of Radiation Oncology, Hospital of the University of Pennsylvania, Philadelphia, PA, USA; e Department of Radiation Oncology, Shaare Zedek Medical Center, Jerusalem, Israel; f Pediatric Palliative Care Unit, Shaare Zedek Medical Center, Jerusalem, Israel; g Department of Anesthesiology, Hadassah Medical Center, Hebrew University of Jerusalem, Jerusalem, Israel; h P-Cure Ltd./Inc, Shilat, Israel

**Keywords:** Pediatric treatments, Proton therapy, Craniospinal irradiation, Upright radiation therapy

## Abstract

Craniospinal irradiation (CSI) is a technically demanding treatment in pediatric oncology, especially for young children, who often require daily anesthesia. Although proton therapy offers dosimetric advantages over photons by eliminating exit dose and reducing exposure to healthy tissues, its global availability is limited due to the cost and complexity of gantry-based systems. Recently, gantry-less proton therapy with upright positioning has recently emerged as a compact, cost-efficient alternative, integrating robotic positioning and vertical CT-based image guidance.

This study reports the first pediatric case of upright proton CSI delivered under intravenous monitored anesthesia care (MAC) using such a system. A four-year-old male patient with relapsed neuroblastoma in the central nervous system received CSI at a dose of 18.0 Gy(RBE) in 12 fractions, along with a simultaneous integrated boost (SIB) of 21.6 Gy(RBE) to the resection cavity. This was followed by a sequential boost to the resection cavity in five fractions, for a total dose of 30.6 Gy(RBE). The treatment was performed in the seated position with customized immobilization, upright volumetric CT verification, and reproducible daily setup. MAC achieved without intubation, allowing continuous airway access.

The patient completed the treatment regimen without interruption, did not experience any grade 2 or higher acute toxicities, and demonstrated adequate tolerance of daily anesthesia. This case demonstrates the clinical feasibility of upright proton CSI under MAC and provides proof of concept for the broader adoption of gantry-less proton therapy in complicated cases.

## Highlights

• First pediatric case of upright proton CSI under daily anesthesia.
• Upright setup enabled unobstructed airway access and safe sedation.
• Daily upright CT imaging ensured reproducible positioning and treatment delivery.

## Introduction

Neuroblastoma is the most common extracranial solid tumor in children, accounting for up to 10 % of pediatric cancers and 15 % of related mortalities [1,2]. In high-risk cases, central nervous system (CNS) metastases occur in 4–8 % of patients and are associated with poor outcomes [3,4]. Craniospinal irradiation (CSI) is employed as part of multimodal salvage therapy to improve disease control in these patients [5,6].

Proton therapy (PT) is an evidence-based modality for pediatric CSI, as recommended by the European Society for Paediatric Oncology (SIOPE). The finite proton range eliminates exit dose and spares anterior organs, thereby reducing acute and late toxicities [7]. However, delivering proton CSI is technically demanding, requiring multiple isocenters and precise field junction matching. While photon therapy has increasingly adopted volumetric image guidance using cone-beam CT or magnetic resonance imaging (MRI), most proton facilities still rely on orthogonal X-rays, limiting soft-tissue verification [8]. Additionally, the conventional proton facilities remain limited by the cost and scale of gantry systems.

Image-guided, gantry-less PT platforms have been developed to overcome these limitations. These systems combine fixed horizontal beamlines with robotic patient positioning and vertical CT-based image guidance, enabling upright treatments that reduce system complexity and cost [9]. Early studies have demonstrated feasibility in adult lung and head and neck cancers [10,11,12]. However, pediatric patients frequently require daily anesthesia, which has not previously been implemented in upright treatments.

Here, we report the first pediatric case of upright proton CSI delivered under daily monitored anesthesia care (MAC) using a gantry-less image-guided system.

## Case

A 4-year-old male patient with high-risk neuroblastoma (stage 4, N-Myc non-amplified, anaplastic lymphoma kinase [ALK] negative) was initially treated with induction chemotherapy, surgical resection, and adjuvant abdominal proton therapy.

On follow-up, a 2-cm right frontal lesion was detected. MRI of the brain and spine showed no leptomeningeal enhancement. The patient underwent gross total resection, and pathology confirmed recurrent neuroblastoma. Considering the high risk of microscopic dissemination despite the absence of radiographic leptomeningeal disease, the multidisciplinary tumor board recommended CSI.

At the start of treatment, the patient was in excellent general condition, with an Eastern Cooperative Oncology Group (ECOG) performance status of 0. He was alert and responsive, with normal neurological development and no focal deficits, gait disturbance, or cranial nerve abnormalities. The incision from the right frontal craniotomy was well healed, with no residual edema or tenderness. His anthropometric measurements included a height of approximately 95 cm and a weight of 16 kg.

## Methods

The treatment was performed at the Sharett Institute of Oncology, Hadassah Medical Center using the novel upright PT platform (P-Cure Proton Therapy System, P-Cure Ltd., Shilat, Israel), that allows for seated irradiation daily intravenous MAC with spontaneous respiration and without intubation.

This PT system integrates a compact synchrotron that delivers pencil beam scanning (PBS) PT. The treatment room is equipped with a fixed horizontal beamline and a six-degrees-of-freedom (6D) robotic positioning chair. Image guidance is facilitated by a vertical CT scanner (Philips Brilliance Big Bore) mounted at a 20° tilt relative to the vertical axis, in conjunction with an orthogonal kilovoltage (kV) X-ray system [9].

**Fig. 1:**
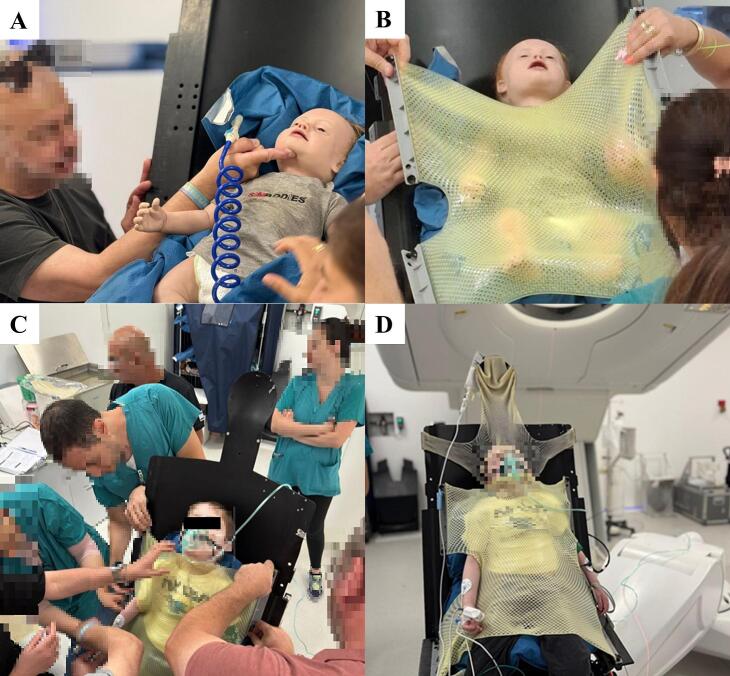
Preparation of immobilization and MAC devices using a doll (A, B). The upright immobilization setup showing forehead and abdominal masks installation with airway access (C) and patient transfer into treatment position after daily imaging (D).

Prior to treatment, the anesthesiology team practiced on a patient-sized doll to simulate the workflow and access the airway (Fig. 1, Fig. 1). During this training a customized immobilization setup was developed to ensure stability during CSI. A vacuum-lock cushion was meticulously shaped beneath the occiput and upper thoracic spine to ensure a straight, extended posture. A custom-molded thermoplastic abdominal mask was applied from the lower chest to just below the chin, providing consistent support to the anterior neck and slight head extension. Cranial stability was further ensured by means of a partial thermoplastic mask positioned over the forehead, thus leaving the nose and mouth unobstructed to facilitate airway access (Fig. 1, Fig. 1).

In-room, vertical CT has been used for treatment planning. The delineation of clinical target volumes (CTVs) for the brain and spine was performed in accordance with international consensus guidelines on craniospinal target volume definition [13]. Vertebral bodies were included following pediatric atlas recommendations [14] to ensure symmetric growth and reduce risk of scoliosis.

**Fig. 2:**
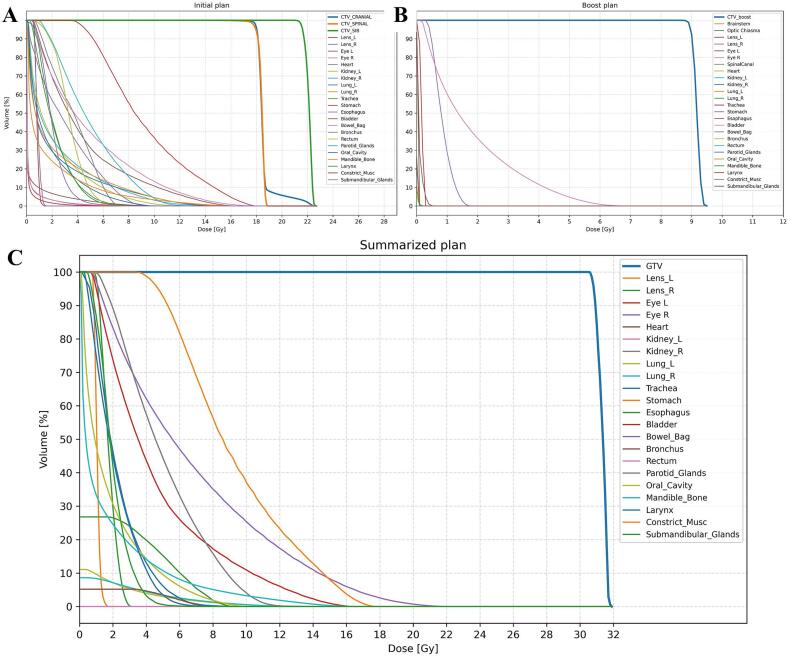
Dose–volume histograms (DVHs) for the initial (A), boost (B) and summarized plans (C).

Proton dose was calculated with a relative biological effectiveness (RBE) of 1.1. Plans were generated in RayStation 2023B. The CSI prescription was 18.0 Gy(RBE) in 12 fractions of 1.5 Gy(RBE). A simultaneous integrated boost (SIB) to the resection cavity was delivered to 21.6 Gy(RBE) in 12 fractions of 1.8 Gy(RBE). A sequential boost plan to the resection cavity, consisting of five fractions of 1.8 Gy(RBE) each, was delivered for a total resection cavity dose of 30.6 Gy(RBE). Dose–volume histograms (DVHs) are shown in Fig. 2.

**Fig. 3:**
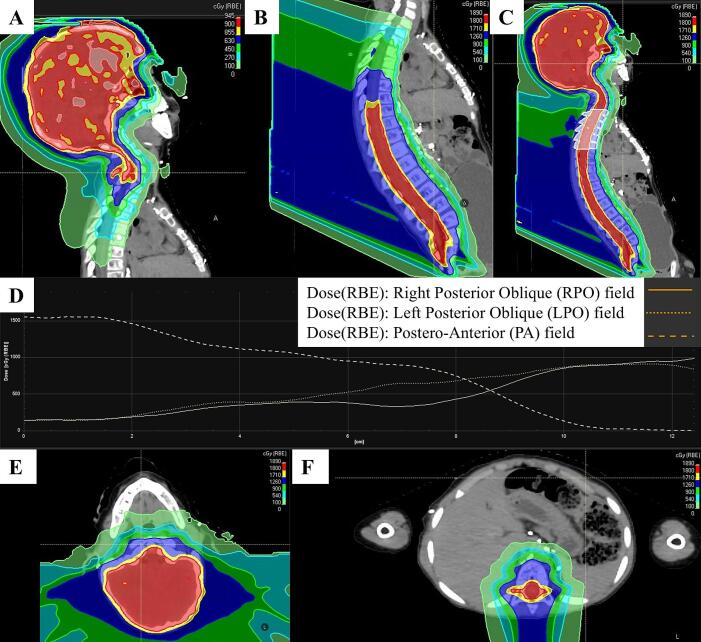
The sagittal dose distributions for LPO (A) and PA (B) fields and the full plan with the junction area highlighted in white (C). The dose profile along the junction showing PA field delivering all dose at ≤ 2 cm, and gradually more dose being delivered by the cranial fields, until all dose delivered at ≥ 10 cm (D). Axial dose distributions illustrate target coverage of the brain with sparing of the oral cavity and pharyngeal constrictor muscles at cranial isocenter (E), and coverage of the spinal canal and vertebral bodies with sparing of the stomach, liver, and bowel at spinal isocenter (F).

Treatment planning employed two isocenters: one for the cranial fields (two beams) and one for the spinal field (single beam), with an intentional overlap region at the craniospinal junction (Fig. 3). The overlap was designed to provide a slow dose gradient over an 8 cm junction area. This optimization technique described by Lin et al. [15] was used and found to be robust to position uncertainties in the longitudinal direction.

**Fig. 4:**
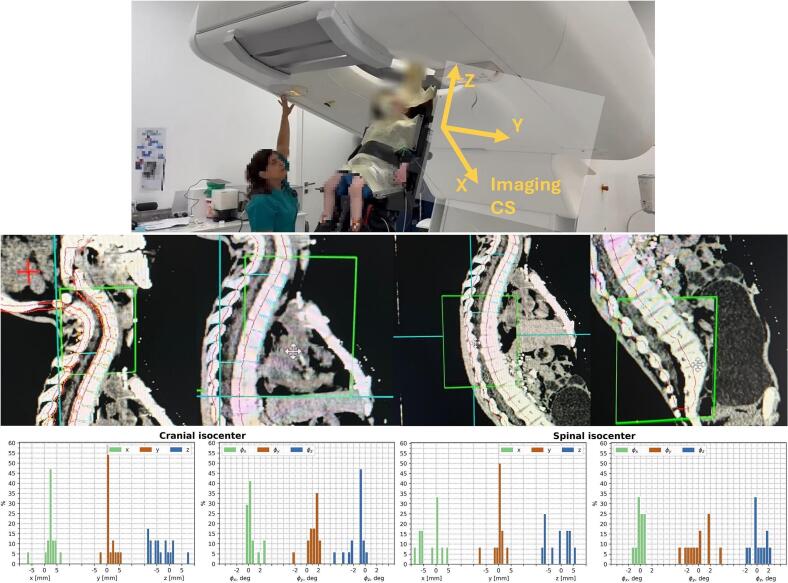
Patient setup for daily 3D/3D registration using the vertical CT scanner (top panel). Example of patient position verification prior to treatment across different regions of the craniospinal axis (middle panel). The area within the green box represents the daily CT data, while the surrounding area shows the reference planning CT. Results of daily 3D/3D image registration for the cranial and spinal isocenters are shown separately (bottom panel). (For interpretation of the references to colour in this figure legend, the reader is referred to the web version of this article.)

The image-guided workflow previously described in the literature [9,11,12] has been implemented. This workflow consists of a daily low-dose 3DCT scan in an upright position and a pair of 2D orthogonal kV X-ray images for each field. In the case described here, there are three fields. Inter‑fraction patient position was verified before every fraction using the daily upright CT, the reference planning CT and 3D/3D image registration (Fig. 4). 2D orthogonal kV X-ray images were used to confirm the position alignment after any manipulation with the chair.

## Results and discussion

The patient underwent CSI and sequential boost without interruption. 6D patient positioning corrections were performed daily based on the 3D/3D registration (Fig. 4). The mean corrective shifts were 〈Δx〉 = 2.38 ± 2.53 mm, 〈Δy〉 = 1.23 ± 1.90 mm, 〈Δz〉 = −3.25 ± 4.49 mm, 〈Δφ_x_〉 = 0.53 ± 0.98 deg, 〈Δφ_y_〉 = 1.09 ± 1.04 deg, 〈Δφ_z_〉 = −1.75 ± 2.00 deg for the cranial isocenter and 〈Δx〉 = −2.10 ± 4.17 mm, 〈Δy〉 = −0.13 ± 2.48 mm, 〈Δz〉 = −1.23 ± 4.72 mm, 〈Δφ_x_〉 = −0.01 ± 0.67 deg, 〈Δφ_y_〉 = 0.01 ± 2.00 deg, 〈Δφ_z_〉 = 0.24 ± 1.22 for the spinal isocenter. All positioning corrections were within the system’s tolerance limits. Average CT dose index (CTDI_vol_) was 33.3 ± 7.5 mGy per daily upright CT. Despite the additional imaging dose, upright volumetric verification was considered mandatory to ensure accurate positioning in this complex geometry. The use of a two-isocenter plan with an intentional overlap region at the craniospinal junction enabled continuous and homogeneous target coverage (Fig. 3).

The total duration of each treatment session (from entering to leaving the treatment room) was 1 h 17 min ± 13 min for the initial plan (first 12 fractions) and 25 min 36 sec ± 5 min for the boost phase (last 5 fractions). This included patient setup, MAC, imaging, verification, and three filed beam delivery. The beam delivery time per field was less than two minutes. As this was the pilot clinical implementation, the radiation therapy technologist (RTT) entered the treatment room and performed all procedures semi-manually under the direct supervision of the medical physicist and anesthesiologist to ensure patient safety. This contributed to the prolonged overall time, particularly during the initial plan.

The upright setup provided unobstructed airway access, enabling safe anesthetic management. The patient tolerated daily anesthesia well, maintained spontaneous respiration, and experienced no complications arising from the sedative agent. No acute toxicities of grade ≥ 2 were observed.

This case highlights several practical workflow considerations:

• *Pillow valve placement:* the pillow valve should be placed on the underside of the chair to prevent interference with head care from the side.
• *Head position*: the patient’s head should be positioned above the body mask adapters.
• Air management: When air is pumped from the pillow, it should not obstruct the mask attachment on the sides.
• *Airway patency:* to maintain patency of the airways, the head should be tilted slightly backward.
• *Mask configuration:* a body mask that secures the chin and sides of the head can be used to achieve fixation, while keeping the airways open and allowing the mask to be placed over the nose and mouth.
• *Hand placement:* at least one hand should remain outside the body mask.

The patient is currently undergoing post-treatment follow-up to assess disease control and late effects. Brain and spine MRI are performed every 2–3 months during the first two years and every 4–6 months thereafter. Systemic surveillance, including abdominal imaging and ^123^I-MIBG scans, is conducted at similar intervals and later extended to annual assessments. Follow-up also includes neurological and developmental evaluations, endocrine testing, and growth monitoring to assess spinal symmetry. Neurocognitive testing and audiometry are initiated one-year post-treatment and repeated annually. Long-term follow-up is planned for at least five years.

## Conclusion

The novel contribution of this report lies in its demonstration, for the first time, of the feasibility of delivering upright proton CSI in a pediatric patient under daily MAC within image-guided workflow. The approach demonstrated reproducible patient setup, accurate beam delivery, and safe anesthetic management. Upright treatment offers workflow and infrastructure advantages, supporting its role as a cost-efficient alternative to conventional proton therapy and expanding access to advanced radiotherapy for children worldwide.

## Declaration of Competing Interest

The authors declare the following financial interests/personal relationships which may be considered as potential competing interests: Alexander Pryanichnikov has a consulting agreement with P-Cure Ltd./Inc. Shimshon Winograd is an employee of P-Cure Ltd./Inc. All remaining authors have declared no conflicts of interest..
